# Supplementary figures and images for: The CLIP-domain serine protease CLIPC9 regulates melanization downstream of SPCLIP1, CLIPA8, and CLIPA28 in the malaria vector Anopheles gambiae
Source: PLoS Pathog. 2020 Oct 12;16(10):e1008985. doi: 10.1371/journal.ppat.1008985 (PMC7580898; doi:10.1371/journal.ppat.1008985)

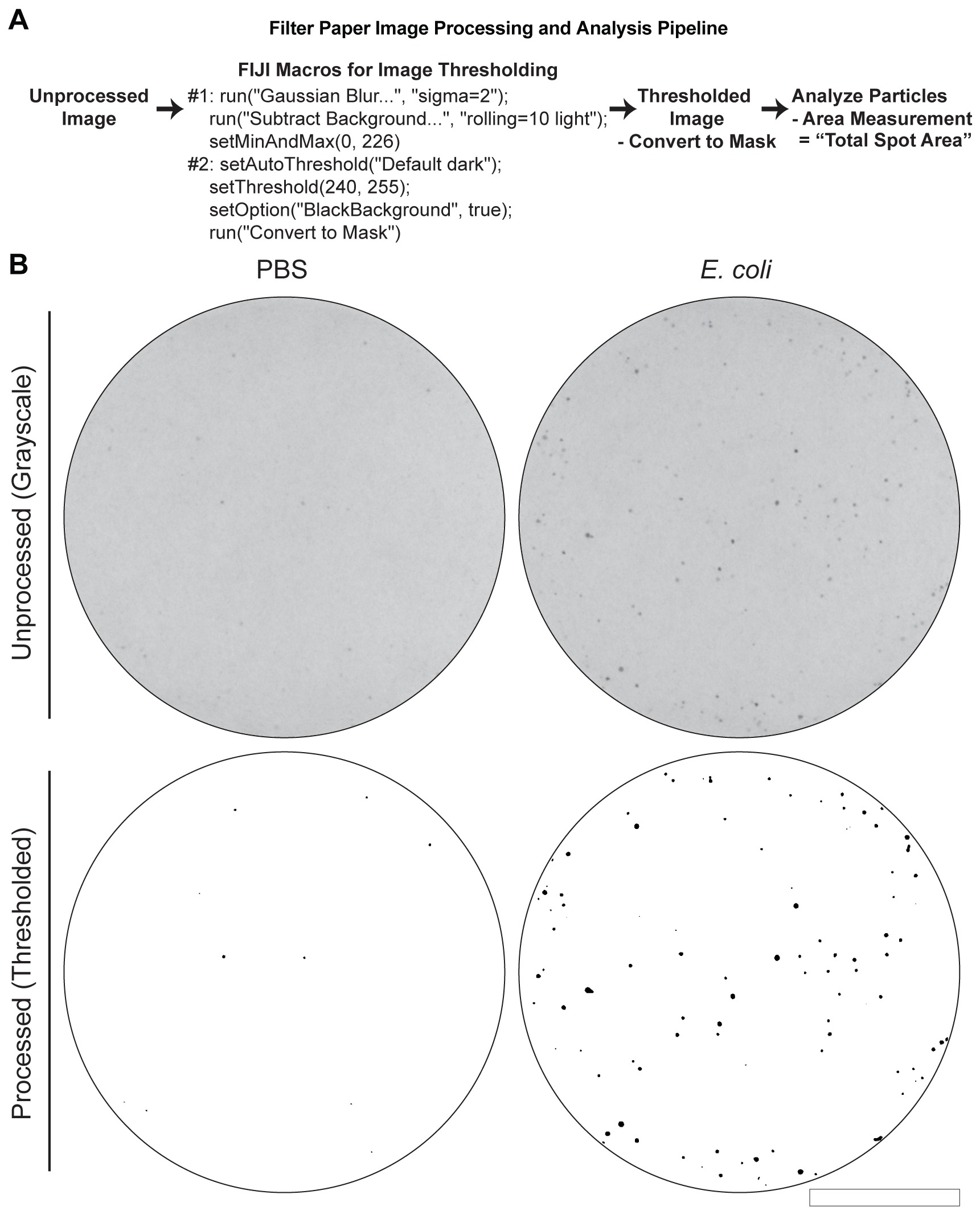

Supplement: S1 Fig — (A) Filter paper image processing and analysis pipeline to generate and quantify thresholded images in FIJI84. (B) Unprocessed (top row, grayscale) and processed (bottom row, thresholded) filter papers from 50 mosquitoes 12 h after PBS or E. coli injection. Scale bar is 2.5 cm. (TIF) [file ppat.1008985.s001.tif]

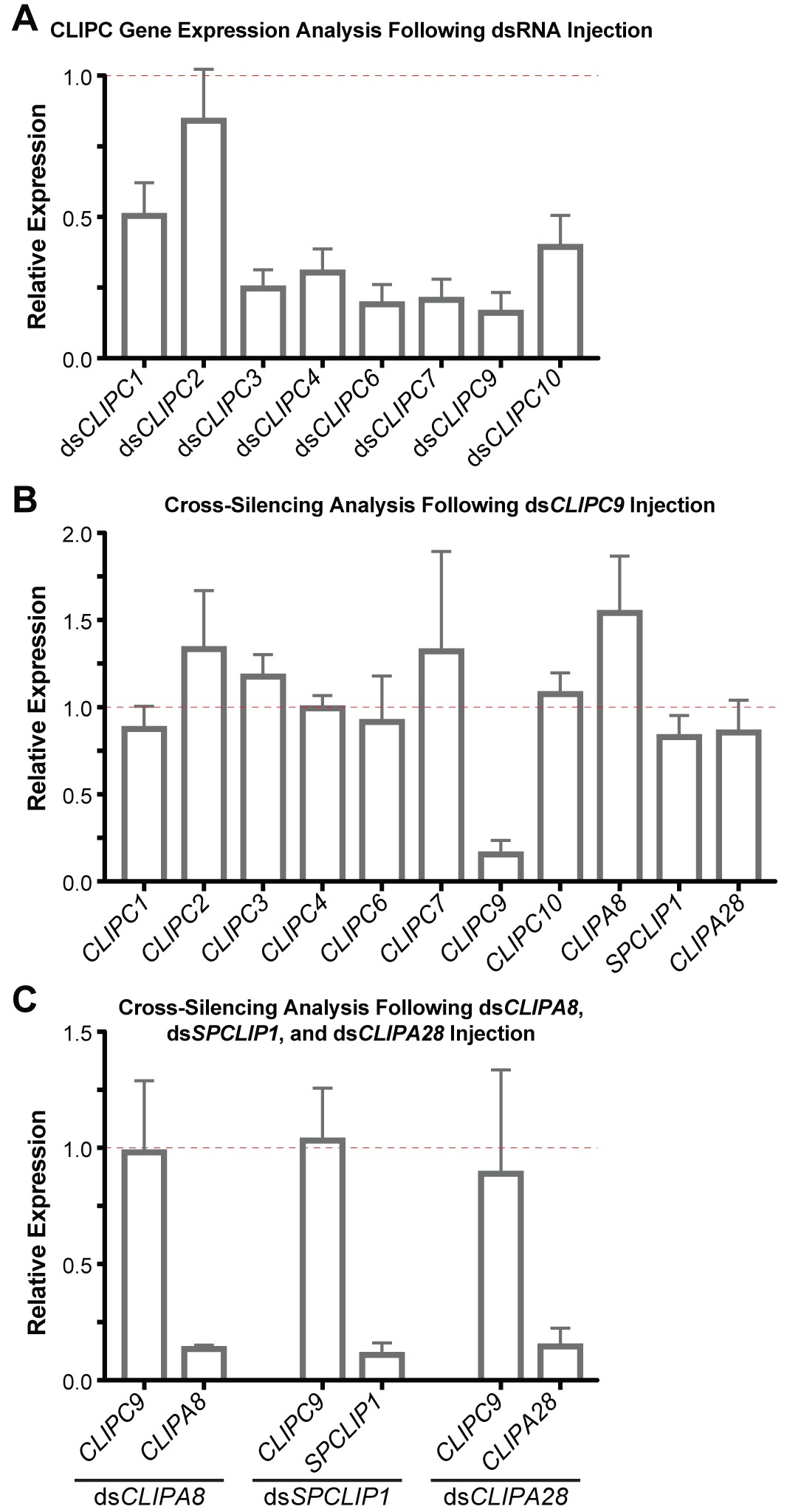

Supplement: S2 Fig — (A) CLIPC screen gene expression analysis 3–4 d after dsRNA administration. (B) Cross-silencing analysis of the CLIPC candidates and the CLIP-SPH pathway members following dsCLIPC9 treatment. CLIPC9 gene expression was included as a positive control. (C) Cross-silencing analysis of CLIPC9 following dsCLIPA8, dsSPCLIP1, and dsCLIPA28 treatments. Expression values for CLIPA8, SPCLIP1, and CLIPA28 were included as positive controls. All mean qRT-PCR expression values are relative to their respective dsLacZ treatment groups, which are set to 1.0 and denoted by the red dotted line. All error bars are ± SD. All data are pooled from three independent biological replicates. (TIF) [file ppat.1008985.s002.tif]

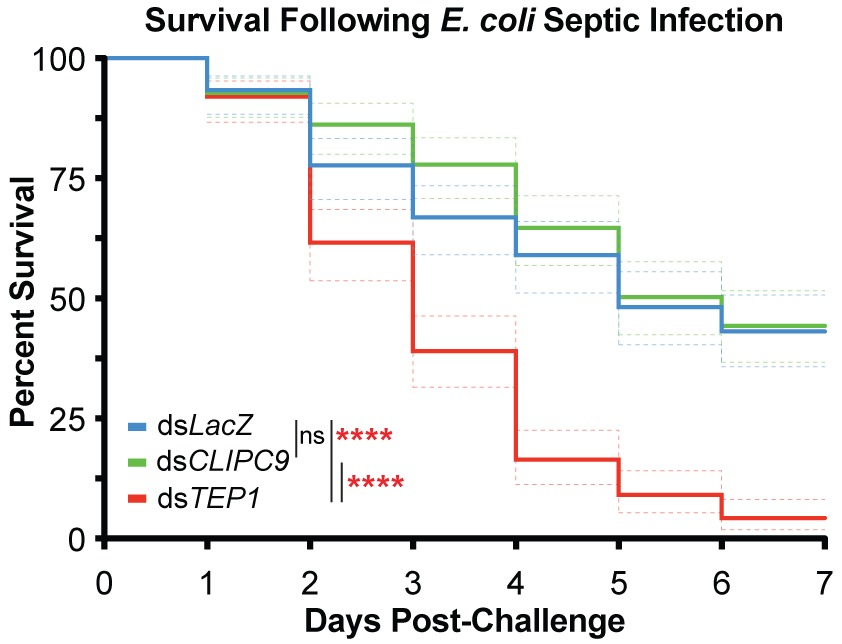

Supplement: S3 Fig — Mosquito survival was tracked for 7 d following septic infection with E. coli at OD 3.2 in dsLacZ-, dsCLIPC9-, and dsTEP1-treated mosquitoes. TEP1 knockdowns were included as a positive control for reduced survival following E. coli infection48. Experimental groups were compared to the dsLacZ group with the Log-rank test. Asterisks denote statistical significance (****p ≤ 0.0001). Curves are averages from three independent biological replicates and dotted lines are 95% confidence intervals. (TIF) [file ppat.1008985.s003.tif]

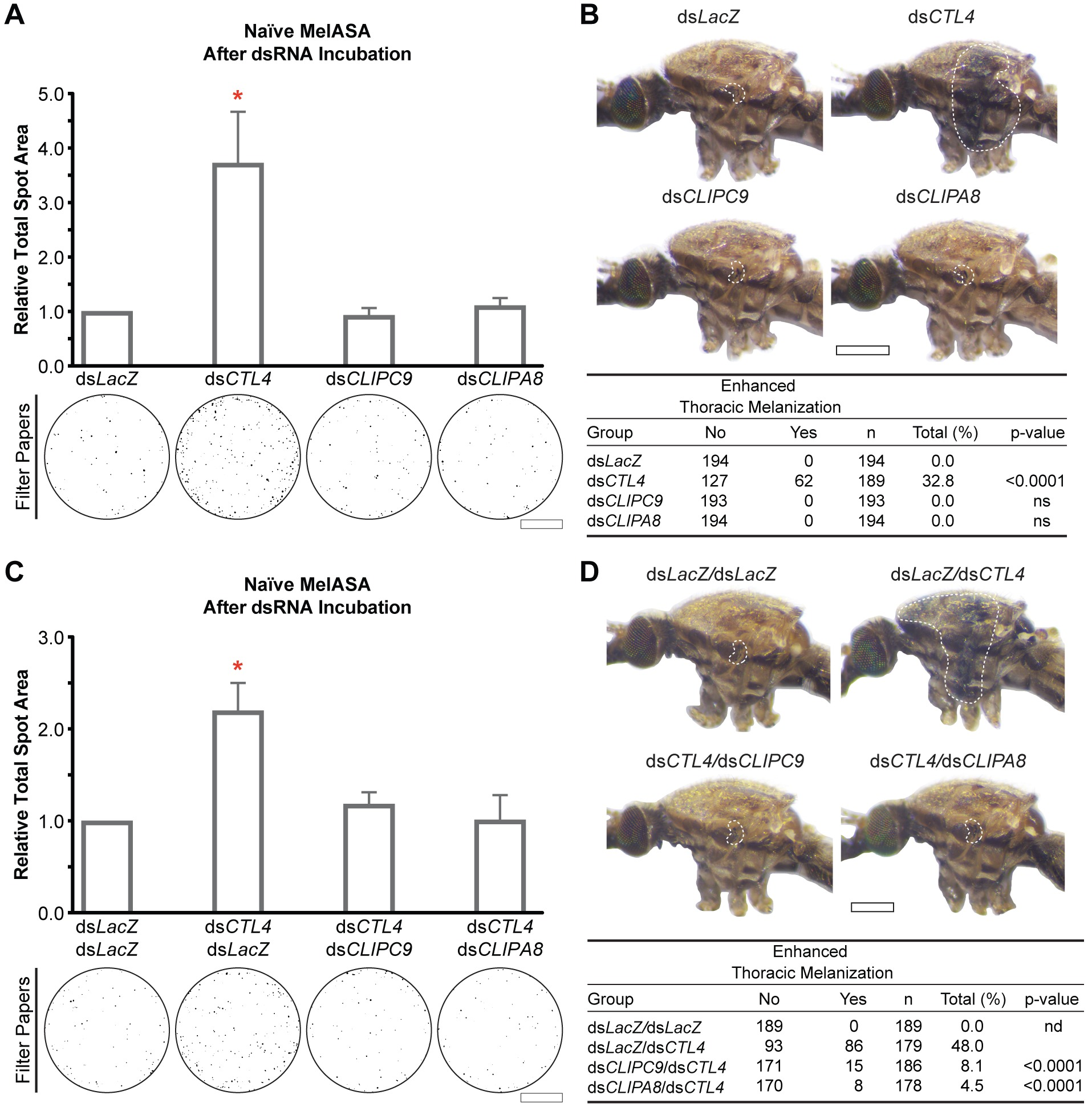

Supplement: S4 Fig — Naïve standard MelASAs performed on filter papers removed from (A) single or (C) double gene knockdown cups 3–4 d after dsRNA injection (one sample t-tests). Final group sizes differed slightly due to typical mosquito attrition, however intraexperimental groups never differed by more than 4 individuals. Error bars are ± SD. Asterisks denote statistical significance (*p ≤ 0.05). Brightfield photomicrographs of mosquito thoraxes 3–4 d after (B) single or (D) double gene knockdown. Single gene knockdown data were compared to dsLacZ control using Fisher’s exact test. The dsLacZ/dsLacZ treatment group was excluded from the double gene knockdown analysis. The dsCLIPC9/dsCTL4 and dsCLIPA8/dsCTL4 treatment groups were each compared to the dsLacZ/dsCTL4 group using Fisher’s exact test. The regions of injection-induced melanization are outlined in white. Scale bars are 2.5 cm in (A) and (C), 500 μm in (B), and 200 μm in (D). All data are compiled from three independent biological replicates. Abbreviations: ns = not significant; nd = not determined. (TIF) [file ppat.1008985.s004.tif]

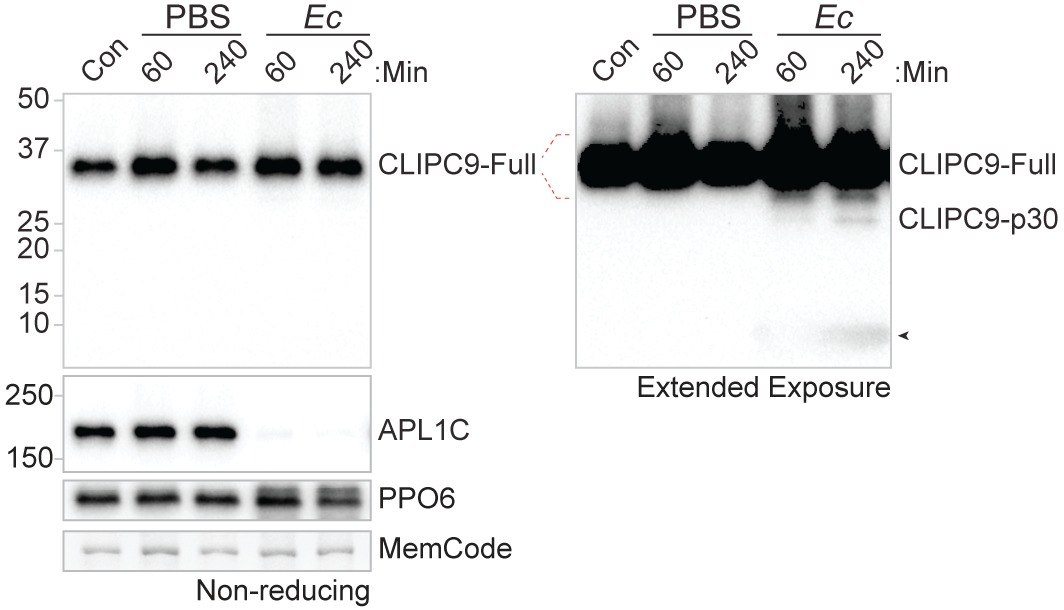

Supplement: S5 Fig — The septic infection-induced cleavage of CLIPC9 was assessed by non-reducing western analysis of naïve/control hemolymph (Con) and hemolymph 60 and 240 min after PBS or E. coli (Ec) challenge. The anti-CLIPC9 extended exposure is provided to the right of the compilation to confirm the absence of the CLIPC9 p12 fragment. Blots were probed with antibodies against APL1C and PPO6 to confirm E. coli exposure and equal protein loading, respectively. Black arrowhead denotes a non-specific haze associated with E. coli injection in western analysis. Membranes were MemCode stained as an additional loading control. Image is representative of three independent biological replicates. (TIF) [file ppat.1008985.s005.tif]

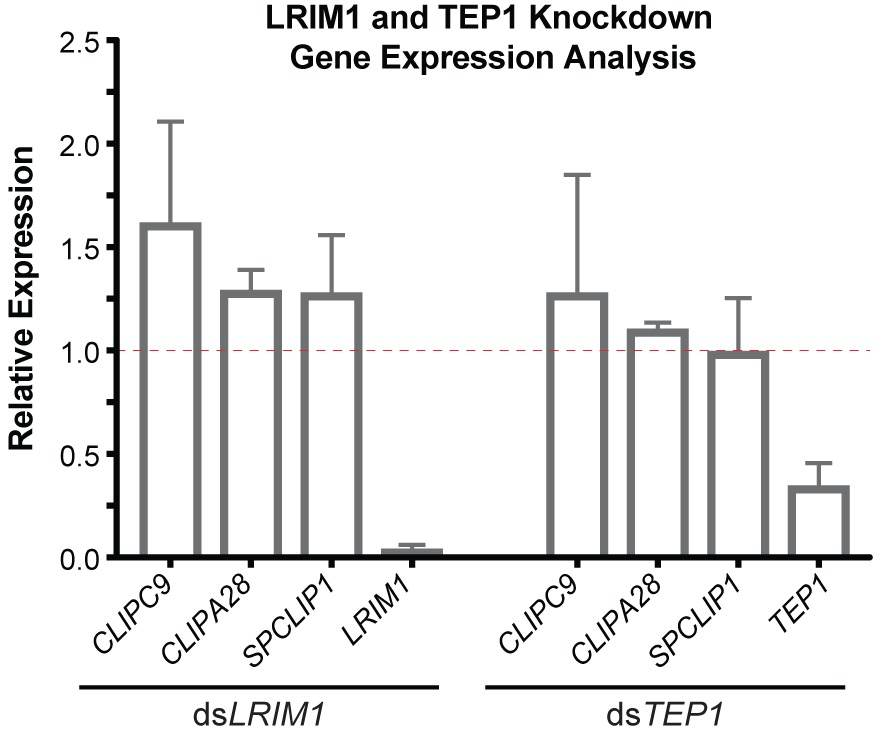

Supplement: S6 Fig — Mean qRT-PCR expression analysis of CLIPC9, CLIPA28, and SPCLIP1 3–4 d after dsLRIM1 and dsTEP1 administration. Expression values for LRIM1 and TEP1 were included as positive controls. Expression values are relative to the dsLacZ treatment group, which is denoted by the red dotted line set to 1.0. Error bars are ± SD. Experimental replicates are from three independent mosquito generations. (TIF) [file ppat.1008985.s006.tif]

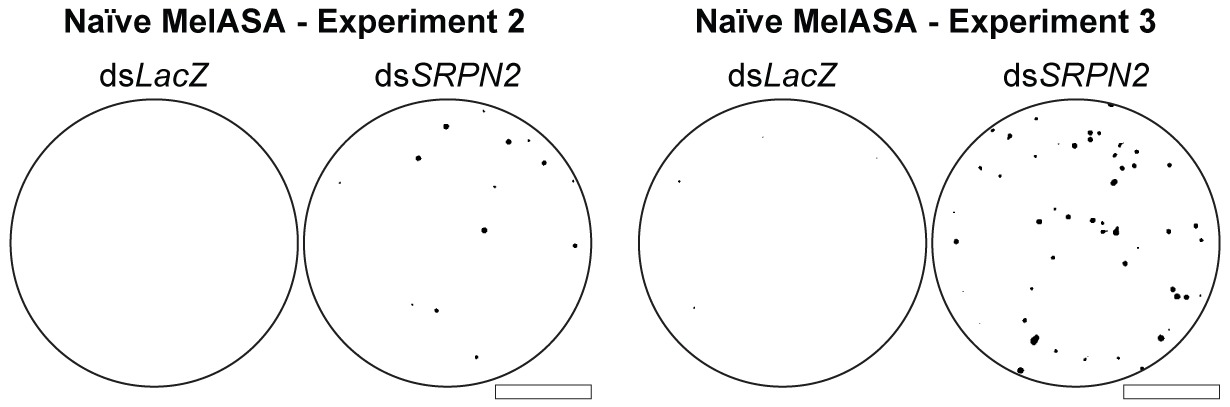

Supplement: S7 Fig — Additional naïve Mini-MelASA replicates 4 d after gene knockdown in dsSRPN2- and dsLacZ-treated mosquitoes. Filter papers were removed after 12 h. Scale bar is 1.3 cm. (TIF) [file ppat.1008985.s007.tif]

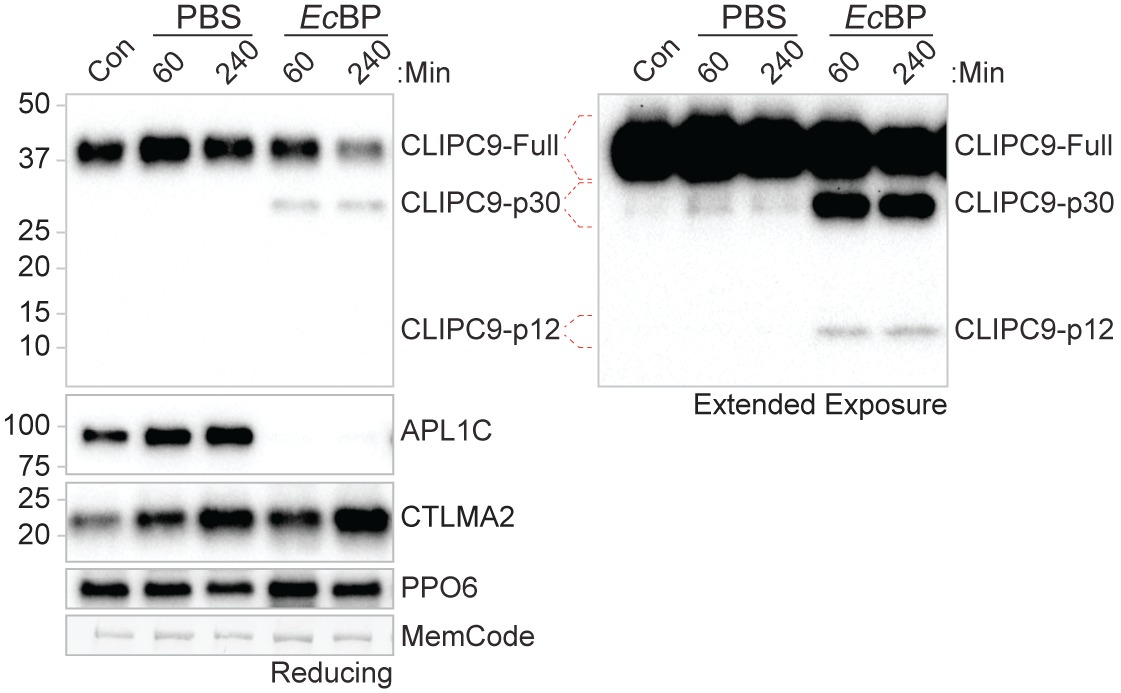

Supplement: S8 Fig — Challenge-induced cleavage of CLIPC9 was assessed by reducing western analysis on whole hemolymph 60 and 240 min after PBS or E. coli bioparticle (EcBP) injection and from naïve/control (Con) mosquitoes. The anti-CLIPC9 extended exposure is provided to the right of the compilation to show detection of the CLIPC9 p12 fragment. Blots were probed against APL1C, CTLMA2, and PPO6 to confirm the EcBP challenge, adequate sample reduction, and equal protein loading, respectively. Membranes were MemCode stained as an additional loading control. Western image is representative of experiments performed on two independent biological replicates. (TIF) [file ppat.1008985.s008.tif]

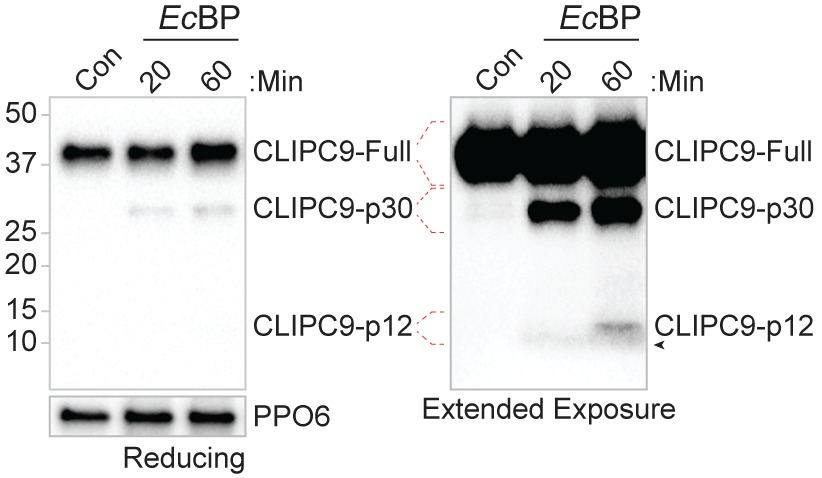

Supplement: S9 Fig — Challenge-induced cleavage of CLIPC9 was assessed by reducing western analysis on whole hemolymph 20 and 60 min after E. coli bioparticle (EcBP) injection and from naïve/control (Con) mosquitoes. The anti-CLIPC9 extended exposure is provided to the right of the compilation to show detection of the CLIPC9 p12 fragment. Blots were probed against PPO6 to confirm equal protein loading. Western image is representative of four independent biological replicates. (TIF) [file ppat.1008985.s009.tif]
