# Supplementary material for: The CLIP-domain serine protease CLIPC9 regulates melanization downstream of SPCLIP1, CLIPA8, and CLIPA28 in the malaria vector Anopheles gambiae
Source: PLoS Pathog. 2020 Oct 12;16(10):e1008985. doi: 10.1371/journal.ppat.1008985 (PMC7580898; doi:10.1371/journal.ppat.1008985)
Supplement: S1 Table — (DOCX) [file ppat.1008985.s010.docx]

| **S1 Table. T7 primers used for dsRNA template synthesis.** | | |
| --- | --- | --- |
| AGAP ID | Name | Sequence |
| n/a | LacZ T7 F | taatacgactcactatagggAGAATCCGACGGGTTGTTACT |
| --- | LacZ T7 R | taatacgactcactatagggCACCACGCTCATCGATAATTT |
| AGAP010731 | CLIPA8 T7 F | taatacgactcactatagggGCAGAACGATGGCTCAGATG |
| --- | CLIPA8 T7 R | taatacgactcactatagggTGCTCGTTGGACGAGTAGAA |
| AGAP010730 | CLIPA28 T7 F | taatacgactcactatagggGAATGGGACATCAGCACCAC |
| --- | CLIPA28 T7 R | taatacgactcactatagggCGCAGGGTGTAGAACGGT |
| AGAP028725 | SPCLIP1 T7 F | taatacgactcactatagggGTCACCGAACACGGCCAAC |
| --- | SPCLIP1 T7 R | taatacgactcactatagggATCGAAGCTGATCGGATCGGG |
| AGAP005335 | CTL4 T7 F | taatacgactcactatagggTGGTTTGATGCCGTGTCCT |
| --- | CTL4 T7 R | taatacgactcactatagggAATAAATTGTCTCGGTTCATCATC |
| AGAP006348 | LRIM1 T7 F | taatacgactcactatagggAATATCTATCTCGCGAACAATAA |
| --- | LRIM1 T7 R | taatacgactcactatagggTGGCACGGTACACTCTTCC |
| AGAP010815 | TEP1 T7 F | taatacgactcactatagggTTTGTGGGCCTTAAAGCGCTG |
| --- | TEP1 T7 R | taatacgactcactatagggACCACGTAACCGCTCGGTAAG |
| AGAP006911 | SRPN2 T7 F | taatacgactcactatagggCGAGGGCGCGGTCATTACG |
| --- | SRPN2 T7 R | taatacgactcactatagggCAGCATTGTTCCGAGGGTTTCATC |
| AGAP008835 | CLIPC1 T7 F | taatacgactcactatagggGAGTATGGGCAGGCTGTGTT |
| --- | CLIPC1 T7 R | taatacgactcactatagggAAGTTTGTGGAAGTTAGGCAGTG |
| AGAP004317 | CLIPC2 T7 F | taatacgactcactatagggGAGACTACCAGAGCGCATCC |
| --- | CLIPC2 T7 R | taatacgactcactatagggGTCGCCAAACTCGTCGTT |
| AGAP004318 | CLIPC3 T7 F | taatacgactcactatagggGCCGACGATCGATAGAAACT |
| --- | CLIPC3 T7 R | taatacgactcactatagggGCAGCGCAAAGAGATCAGAC |
| AGAP000573 | CLIPC4 T7 F | taatacgactcactatagggGAGTTCAGCGGCAACGAT |
| --- | CLIPC4 T7 R | taatacgactcactatagggTGCACCTGCTTGATCTCGTA |
| AGAP000571 | CLIPC5 T7 F | taatacgactcactatagggACGTACGATGGGACGCAG |
| --- | CLIPC5 T7 R | taatacgactcactatagggAGCAGGAAGCGAACCGTTAT |
| AGAP000315 | CLIPC6 T7 F | taatacgactcactatagggCAGACGCTTTACGAGGGAGA |
| --- | CLIPC6 T7 R | taatacgactcactatagggTACTTGGGCACCTGTTCGC |
| AGAP003689 | CLIPC7 T7 F | taatacgactcactatagggCAATTACGGTACAGCTGGGAA |
| --- | CLIPC7 T7 R | taatacgactcactatagggCCTCCGTTAGCGTAAGGTTG |
| AGAP004719 | CLIPC9 T7 F | taatacgactcactatagggGGTGCAGTAAGAAGGCCCAT |
| --- | CLIPC9 T7 R | taatacgactcactatagggACTGCATGTCCAAGCAATCC |
| AGAP000572 | CLIPC10 T7 F | taatacgactcactatagggCTCATCTCGTCCCGGTTTCT |
| --- | CLIPC10 T7 R | taatacgactcactatagggGCGATATCGTTCTGGTACGTG |
