# Supplementary material for: The CLIP-domain serine protease CLIPC9 regulates melanization downstream of SPCLIP1, CLIPA8, and CLIPA28 in the malaria vector Anopheles gambiae
Source: PLoS Pathog. 2020 Oct 12;16(10):e1008985. doi: 10.1371/journal.ppat.1008985 (PMC7580898; doi:10.1371/journal.ppat.1008985)
Supplement: S2 Table — (DOCX) [file ppat.1008985.s011.docx]

| **S2 Table. Primers used for qRT-PCR analysis.** | | |
| --- | --- | --- |
| AGAP ID | Name | Sequence |
| AGAP010592 | S7 qPCR F | GTGCGCGAGTTGGAGAAGA |
| --- | S7 qPCR R | ATCGGTTTGGGCAGAATGC |
| AGAP010731 | CLIPA8 qPCR F | GATCGATTCGACGACCAACT |
| --- | CLIPA8 qPCR R | GCAGGTCGACTCGCTTTAAC |
| AGAP010730 | CLIPA28 qPCR F | ATAAAGCATGCCCAAACCAC |
| --- | CLIPA28 qPCR R | CTGACAGCACACCAGCAGAT |
| AGAP028725 | SPCLIP1 qPCR F | CTTGCTGAACGACGACGATA |
| --- | SPCLIP1 qPCR R | TCCTGTTCCTCCTCGTCACT |
| AGAP005335 | CTL4 qPCR F | GCACGGGTACAGGGCTACTA |
| --- | CTL4 qPCR R | GCGTGGTGTACAGCTTTCCT |
| AGAP006348 | LRIM1 qPCR F | CATCCGCGATTGGGATATGT |
| --- | LRIM1 qPCR R | CTTCTTGAGCCGTGCATTTTC |
| AGAP010815 | TEP1 qPCR F | AAAGCTGTTGCGTCAGGG |
| --- | TEP1 qPCR R | TTCTCCCACACACCAAACGAA |
| AGAP006911 | SRPN2 qPCR F | CCATACGCGCAGCTACTACA |
| --- | SRPN2 qPCR R | ACCTCGATGAAGTCGTCCAC |
| AGAP008835 | CLIPC1 qPCR F | CACCCCGAGTACAAGCAAAC |
| --- | CLIPC1 qPCR R | CGTGCGTAGGGTGAGAAGAT |
| AGAP004317 | CLIPC2 qPCR F | CTCGTTCGGTATTGGCTGTG |
| --- | CLIPC2 qPCR R | TGCCCTCGATCCAGTCTATG |
| AGAP004318 | CLIPC3 qPCR F | CCCATCGGAAGTGCTAAACG |
| --- | CLIPC3 qPCR R | TGGCACCAAGATACTCCGTT |
| AGAP000573 | CLIPC4 qPCR F | ATTGGACCGTCGATCAACCT |
| --- | CLIPC4 qPCR R | GTGCCCTTCATCAGCTTGTC |
| AGAP000315 | CLIPC6 qPCR F | AAGGGTACGGCATCATCGAT |
| --- | CLIPC6 qPCR R | TGGTTACACTTTTGCCACGG |
| AGAP003689 | CLIPC7 qPCR F | CTATTGTCACGTTGCGCTCA |
| --- | CLIPC7 qPCR R | GATTTCCAGTCTTCCGGCAC |
| AGAP004719 | CLIPC9 qPCR F | ATTGCAATCCGGTGAACATT |
| --- | CLIPC9 qPCR R | TGGGTTCGAGATGAAGCTCT |
| AGAP000572 | CLIPC10 qPCR F | GAGCAGAACGAAACGGTGG |
| --- | CLIPC10 qPCR R | CGTGATGCCGACCAGATAGT |
