# Supplementary material for: The CLIP-domain serine protease CLIPC9 regulates melanization downstream of SPCLIP1, CLIPA8, and CLIPA28 in the malaria vector Anopheles gambiae
Source: PLoS Pathog. 2020 Oct 12;16(10):e1008985. doi: 10.1371/journal.ppat.1008985 (PMC7580898; doi:10.1371/journal.ppat.1008985)
Supplement: S3 Table — (DOCX) [file ppat.1008985.s012.docx]

| **S3 Table. Cloning primers used for protein production.** | | |
| --- | --- | --- |
| AGAP ID | Name | Sequence |
| AGAP004719 | CLIPC9-FL BamHI F | ﻿CATGGATCCGCAGTAAGAAGGCCCATTAATATAC |
| --- | CLIPC9-FL HindIII R | ﻿CATAAGCTTTTAGTTATCCCTAGTAGCACTAAC |
| AGAP004719 | CLIPC9-FL NcoI F | GATACCATGGCAGTAAGAAGG |
| --- | CLIPC9-FL XhoI R | CGTTCTCGAGTTAGTTATCCCTAGTAG |
| AGAP010730 | CLIPA28-FL BamHI F | ﻿CATGGATCCCAAGACATTGAAGAAGAACTGAGATG |
| --- | CLIPA28-FL HindIII R | ﻿CATAAGCTTTTACAATTTTATATCAAAACTCTC |
