# Supplementary material for: The CLIP-domain serine protease CLIPC9 regulates melanization downstream of SPCLIP1, CLIPA8, and CLIPA28 in the malaria vector Anopheles gambiae
Source: PLoS Pathog. 2020 Oct 12;16(10):e1008985. doi: 10.1371/journal.ppat.1008985 (PMC7580898; doi:10.1371/journal.ppat.1008985)
Supplement: Attachment — Submitted filename: Responses_to_Editor_and_Reviewers.docx [file ppat.1008985.s014.docx]

Response to the editor and reviewers for manuscript PPATHOGENS-S-20-01690 **The CLIP-domain serine protease CLIPC9 regulates melanization downstream of SPCLIP1, CLIPA8, and CLIPA28 in the malaria vector *Anopheles gambiae***

We thank the editor and reviewers for their insightful comments and suggestions for improving the manuscript. We have incorporated all comments and made all corrections suggested by the reviewers as detailed below. Comments are in blue and our responses in black. Line numbers refer to the revised manuscript.

In one case, our response required addition of new control data, which we have provided as two new panels in Supplemental Fig. 4. Two reviewers had suggestions concerning our comprehensive model. Reviewer 1 wanted the model to include all proteins from the study and reviewer 2 suggested being more cautious about implying definitive placement of pathway components, like LRIM1/APL1C and TEP1. We have revised the final model in Fig. 10D to include additional proteins from the study and made changes to the text to address both of the reviewers’ points (see below).

We note that to address all comments we have uploaded revised versions of the following figures and their associated legends (full details provided below):

Main figures: 2, 6 and 10. Supporting figures: S4, S5, S8, and S9.

Your manuscript was evaluated by three reviewers and me. The clear consensus is that this manuscript is a significant scientific contribution because of (i) its mechanistic discoveries on the melanization pathway of a mosquito that is a major vector of malaria in Africa, and (ii) its presentation of a novel, non-invasive method for measuring phenoloxidase activity in mosquitoes.

When revising your manuscript, please pay attention to the reviewer comments.

Reviewer 1 requests elaboration on the CLIPC9 RNAi survival phenotype. The manuscript already draws parallels to what has been published for CLIPA8, but elaboration on this point is warranted. Reviewer 1 also recommends an experiment that you should keep in mind when conducting follow-up studies (so, not required for this submission).

We have expanded the discussion of the CLIPC9 survival phenotype on lines 562-66 (complete response below). We also agree with the reviewer about the suggestion to include antibiotic treatment in future work to determine if the injection-site melanization observed in CTL4 knockdowns is caused in response to an opportunistic infection or tissue damage. We have discussed this on lines 245-47, and we will certainly keep this in mind for our follow-up experiments. Thanks!

Reviewer 2 presents some technical questions on spontaneous melanization and several Western blots, plus the strength of the model. I believe all of these issues can be addressed in the revised manuscript and rebuttal letter (more on this below).

We have provided responses to all of the questions from reviewer 2 concerning the relationship between spontaneous, tissue, and microbial melanization. We checked and updated the manuscript to confirm specific terms like “melanization immune response”, “microbial melanization”, “spontaneous melanization”, and “tissue melanization” are used where appropriate. We have also revised the presentation of the westerns showing CLIPC9 cleavage to address the reviewer’s comment, which we believe improves the clarity of the data and its interpretation (complete responses below).

Reviewer 3 asks for further explanation or clarification on some aspects of the interpretation of the data. Note that reviewers 2 and 3 shared some concern about the interpretation of CLIPC9 cleavage, based on the p12/p30 Western blots. Clarifying this point will be essential in your revision.

We have clarified the presentation of the data and interpretation to address both the exposure time and interpretation of CLIPC9 cleavage (complete response below). We agree with the points raised here and believe that the revised western figures improve the manuscript.

In addition, I have several minor comments. On more than one occasion the manuscripts extrapolates the E. coli findings to all Gram(-) bacteria (for example, lines 181-182, 193). This claim is not warranted and should be deleted. To make this claim, the study would have to examine several Gram(-) bacteria and obtain similar findings for all of them.

We agree that our data should not be extrapolated to all gram-negative bacteria. We have modified the statements on lines 52-53, 191-92, 205, 638, and 678-79 to specifically address *E. coli*, rather than gram-negative bacteria.

I also recommend that you not use the term “master regulator” (as in the submission letter) or “master negative regulator” (line 330-331) because master regulators are classically defined as transcription factors related to cell differentiation.

We have removed the reference to SRPN2 as a ‘master negative regulator’ on lines 366-67 to avoid confusion with the more familiar usage of this term.

Finally, multiple statistical tests were used, and they are clearly detailed in the figure legends. I imagine that they were selected after testing the data for normality. If that is the case, include a sentence to that effect in the “statistical analyses” section of the methods. If that is not the case, then explain how the tests were selected.

We thank the editor for identifying this omission. Indeed, the decision to proceed with parametric statistical analyses was preceded by a confirmatory assessment of normality. Non-parametric statistical analyses were used when data were not normally distributed (*i.e.* viable oocyst and melanized ookinete counts). The rationale for our selection of statistical tests has been added to the Materials and Methods in lines 766-69.

Reviewer Comments (if any, and for reference):

Reviewer's Responses to Questions

**Part I – Summary**

Please use this section to discuss strengths/weaknesses of study, novelty/significance, general execution and scholarship.

Reviewer #1: This manuscript described the development of new methodology for the study of mosquito factors involved in melanization, and the use of this method to functionally characterize CLIPC9 and other factors in the mosquito's melanization responses to bacteria and Plasmodium. The study involves complex mechanistic analyses that generate new knowledge on multiple factors of the mosquito's complement and melanization-based responses to infection and possibly also injury.

The provided model provided in figure 10D is somewhat limited and could be expanded with the additional factors that are being addressed in this study to provide a more complete picture to the reader: basically, an effort should be made din integrating the models provided in the various figures.

We thank reviewer 1 for this feedback on how to improve our model by integrating all the findings from this study. We have submitted a revised Fig. 10D and legend. We have positioned the CLIP-SPH pathway and CLIPC9 proteolysis downstream of mosquito complement to integrate our finding in Fig. 7, which suggests that CLIPC9, similar to SPCLIP1 and CLIPA28, is regulated downstream of TEP1. We have also included the CTL4/CTLMA2 heterodimer in our model to include our identification of CTLMA2 on *E. coli* surfaces in Fig. 10B-C and our recent characterization of it as a negative regulator of *E. coli*-induced PO activity.

Reviewer #2: Sousa et al. report in this manuscript the involvement of Anopheles gambiae CLIPC9 in melanization, a mosquito immune response against malaria parasites, microbial pathogens, and sometimes spontaneously. While mechanism is unclear, a non-destructive method was devised and shown to be a good substitute for the phenoloxidase assay, which is challenging for small insects with a limited amount of hemolymph. Combining the new technique with RNAi screening of the CLIPC genes, alone or in conjunction with one of the CLIPA, TEP1 and other genes, they identified CLIPC9 as the first clip-domain serine protease in group C participating in melanization and discovered its relationships with TEP1, SPCLIP1, CLIPA8, CLIPA28, and SRPN2. These connections are indirect but built on previous studies in this and other insects. The authors also found CLIPA8 is needed for CLIPC9 localization on E. coli bioparticles. The experiments were well deigned and the results were properly interpreted along with inspiring discussions. While the significance and quality of this research have met the standards of PLoS Pathogens, I suggest the authors to improve their manuscript by addressing several questions.

No questions were listed in this section. Questions by reviewer 2 addressed below where they appear.

Reviewer #3: The manuscript by Sousa et al is a well-executed study of mosquito melanization, describing an innovative new method to examine melanization using the melanization-associated spot assay, and in utilizing this assay to implicate CLIPC9 in the melanization pathway of An. gambiae. The manuscript is well-written and the results are straight-forward, thorough, and well-presented. Overall, I think it is a great study, and have only minor comments that can be easily corrected in a revised manuscript.

No questions were listed in this section. Questions by reviewer 3 addressed below where they appear.

**Part II – Major Issues: Key Experiments Required for Acceptance**
Please use this section to detail the key new experiments or modifications of existing experiments that should be absolutely required to validate study conclusions.

Generally, there should be no more than 3 such required experiments or major modifications for a "Major Revision" recommendation. If more than 3 experiments are necessary to validate the study conclusions, then you are encouraged to recommend "Reject".

Reviewer #1: (No Response)

Reviewer #2: In the Abstract, the authors stated that their method is based on quantitation of infection-induced excreta. In Fig. 9A and Fig. S7, spontaneous melanization also generate brown spots. How can I be sure if RNAi induced change in melanization is not associated with spontaneous melanization? A systematic check and presentation of additional controls as supplemental materials may be needed to address this concern.

We thank reviewer 2 for these comments and question. We agree that it is more accurate to state that MelASA is based on the quantitation of melanotic excreta, as this encapsulates its utility to identify melanization components after microbial infection or following dsRNA treatment-induced melanization (*i.e.* ds*CTL4* and ds*SRPN2*). We have modified the Abstract line 28 accordingly.

Reviewer 2 points out that spontaneous melanization following ds*SRPN2* treatment leads to an increase in spot area from melanotic excreta. Indeed, we were excited by this finding, as it confirmed the ability of MelASA to identify negative regulators of the melanization cascade. We appreciate the reviewer’s comment that spontaneous melanization following dsRNA treatment could potentially confound the interpretation of MelASAs performed following *E. coli* septic infection. In fact, because of this, we would strongly advocate testing any gene whose silencing phenotype led to a significant MelASA increase following *E. coli* septic infection for increased baseline/spontaneous melanization cascade activity following gene silencing. Had increased MelASA been observed for any of the screened CLIPC candidates we would have performed such controls. Regardless, given that we did not perform baseline MelASA analyses on all CLIPC candidates we modified our description of the CLIPC9 MelASA result in Fig. 3A on line 177 to indicate that it was the only candidate with a significant effect on MelASA.

To specifically address the reviewer’s point and to provide a baseline control for CLIPC9 we have provided additional MelASA analyses on filter papers left in the housing cups during the 3-4 d period following ds*LacZ-*, ds*CLIPC9*-, ds*CLIPA8*-, and ds*CTL4* treatments (See revised Fig S4 and lines 227-31). As expected, these experiments revealed no significant difference in baseline MelASA following ds*CLIPC9* and ds*CLIPA8* treatments, but a 3.7-fold increase in total spot area 3-4 d after ds*CTL4* administration. In fact, observations of the increased spot area on these papers is what initially led to the identification of the ds*CTL4*-induced thoracic melanization phenotype. For brevity, these data were excluded from the initial submission. Moreover, these analyses support the requirement of CLIPC9 and CLIPA8 in ds*CTL4*-induced thoracic melanization (See lines 237-42). These data show that silencing CLIPC9 or members of the CLIP-SPH pathway that we tested do not cause dsRNA injection/spontaneous melanization-induced increases in MelASA and highlight the ease in detecting such phenotypes.

In Fig. 6B (Fig. S8 and Fig. S9), Standard exposure, is CLIPC9-p12 cropped from the image of extended exposure by mistake? Otherwise, I couldn’t explain why there is no major increase in the 12 kDa band intensity, which is apparent for CLIPC9-p30. Note the intensity ratio of the two bands are high on the right. Based on the ratio, I think the authors need to indicate in the figure legends to Fig. 8A-C, Fig. S8, and Fig. S9 whether or not the strips CLIPC9-p12 came from prolonged exposure. How long were the standard and extended exposures?

We thank reviewer 2 for highlighting this as it was unclear. The reason the Fig. 6B CLIPC9 p12 fragment intensity did not increase between the standard and extended exposures is because it was intentionally cropped from the extended panel. The CLIPC9 p12 fragment was consistently evident following extended blot exposures and the presented strips in Figs 6B, 8A-C, S5, S8, and S9 came from extended exposures. We appreciate the reviewer’s confusion and have decided to make this clearer by not combing standard and extended exposures in the Figs 6B, S5, S8, and S9 compilations. Revised versions of Figs 6, S5, S8, and S9 and their legends are provided. The text was modified on lines 266-69, and 1161-62. We have explicitly stated the origins of all CLIPC9 p12 strips in Fig. 8A-C on lines 1180-81, which come from their respective anti-CLIPC9 extended exposures. In addition, the average CLIPC9 exposure times to detect the full-length/p30 bands and p12 band are now included in the Materials and Methods lines 746-48.

Reviewer #3: There is nothing that I consider to be a major issue. No further experiments needed.

We thank the reviewer for this feedback.

Part III – Minor Issues: Editorial and Data Presentation Modifications
Please use this section for editorial suggestions as well as relatively minor modifications of existing data that would enhance clarity.

Reviewer #1: The lack of mortality and bacterial melanization in CLIPC9 mosquitoes should be explained and elaborated on. Is the CLIPC9 -regulated defense reaction a minor components of the defense or a post-killing step.

We believe that the importance of the melanization response in immune defense is likely microbe-specific. There is convincing evidence for this in *Drosophila* and it is likely the case in *An. gambiae* given the requirement of CLIPA8 and CLIPA28 for defense against the entomopathogenic fungus, *B. bassiana*, but not bacteria tested to date. We speculate that CLIPC9 silencing may also reduce resistance to *B. bassiana,* and we are actively exploring this possibility. We have discussed these points on lines 562-66. Thus, the reviewer rightly points out that the lack of an *E. coli* challenge survival phenotype in CLIPC9 knockdowns may be due to melanization being a minor contributor to defense against this bacterium.

The injection-site melanization of dsCTL4 treated mosquitoes is interesting and the authors hypothesize that it could be a result of local infection. That could have been tested using antibiotic treatment.

Great suggestion. We agree with this approach for determining if ds*CTL4*-induced thoracic melanization is caused by a local infection and have mentioned this on lines 245-47. We plan to further characterize this phenotype in future experiments and will be sure to include antibiotic treatments in these analyses.

Reviewer #2: TEP1 cleavage is mediated by a specific proteolytic enzyme in plasma, not necessarily but likely a serine protease. As such, is it theoretically correct to place LRIM1-APL1C-TEP1 on top of the serine protease-serine protease homolog system? I suggest the authors to tune down the current model by emphasizing its tentativeness. At least, not describing TEP1 as “the most upstream one” of the mosquito melanization cascade. Thinking more about the model’s limitations may help to open up ways to overcome them.

We agree with reviewer 2 that caution should be exercised when describing the position of TEP1 in the melanization cascade. TEP1_cut_, which participates in a ternary complex with LRIM1/APL1C is often depicted or described to be upstream of the CLIP-SPHs required for melanization (Povelones et al., 2013; El Moussawi et al., 2019). This is due to the requirement of TEP1 and LRIM1/APL1C for microbial melanization (Blandin et al., 2004; Osta et al., 2004; Yassine et al., 2014), the requirement of TEP1 for the localization of PPO6 (Yassine et al., 2011) and SPCLIP1 to microbial surfaces (Povelones et al., 2013), and the requirement of TEP1 for the cleavage of both CLIPA8 (Povelones et al., 2013) and CLIPA28 (El Moussawi et al., 2019). Despite this growing consensus, the reviewer correctly points out the value of considering the limitations of biological models. To specifically address the reviewer’s comments, we have modified the phrase that “TEP1 is the most upstream *An. gambiae* melanization cascade component identified to date…:” to “TEP1 functions upstream in the *An. gambiae* melanization cascade” on line 294. We have also emphasized the tentativeness of our model by including the term ‘Working model’ in the title of the Fig. 10D panel, which accompanies our original description of our Fig. 10D model as a ‘Working model…’ on lines 1215-16.

I am a little confused by Fig. 5. Are they discussing about “tissue melanization” or “immune melanization” here and the rest of the paper? I understand the definitions were made by other people, not perfectly clear but useful. I would like to see how authors of this manuscript consider their results in relation to the two concepts.

We thank reviewer 2 for this question and are happy to provide clarity. Figure 5 demonstrates that CLIPC9 is required for *P. berghei* ookinete melanization, but not killing in the CTL4/CTLMA2 knockdown background. Thus, Fig. 5 addresses the melanization immune response. Self-tissue melanization is presented following SRPN2 silencing in Fig. 9A and potentially around the ds*CTL4* injection site in Fig. S4. While we show that SRPN2 knockdown enhances CLIPC9 cleavage, determining its role in self-tissue melanization/melanotic pseudotumor formation is beyond the scope of this manuscript. Furthermore, while we do show that both CLIPA8 and CLIPC9 are required for the ds*CTL4*-induced thoracic melanization response, we are actively working to determine the cause of this melanization (*e.g.* is it melanization of wounded tissue or microbial melanization?). The contribution of mosquito complement, components of the CLIP-SPH pathway, and CLIPC9 toward the melanization of self-tissue awaits characterization, and we are quite curious to see to what degree (if any) microbial and tissue melanization are distinct.

To make this distinction clearer, we have made adjustments in the Abstract on lines 25, 41; the Introduction on lines 114-17; the Results on lines 128, 135, 152, 166, 167-68, 187, 239-42, 328, 349-50; and the Discussion on lines 415-417, 422-423, 430, 571-72. We have also modified Fig. 2H to include the term “*E. coli* Septic Infection” to clearly link this figure with microbial melanization.

Reviewer #3: -Regarding the results of Figure 5, some additional explanation is needed in the results section to explain differences in melanization and oocyst phenotypes for the double-silencing experiments for CLIPC9/CTL4 and CLIPA8/CTL4. While both CLIPC9 and CLIPA8 are able to negate the influence of CTL4 on melanization, it is unclear why when live oocysts are examined, only CLIPA8-silencing is able to negate the influence of CTL4-silencing on oocyst numbers. The reason for this is unclear.

We do not know the reason for the lack of an oocyst recovery in the ds*CTL4*/ds*CLIPC9* group. This finding was unexpected given the virtually indistinguishable *E. coli* melanization phenotypes shared between CLIPC9 and CLIPA8. We believe it to be either a consequence of the slightly less potent block in ookinete melanization in ds*CTL4*/ds*CLIPC9* treated mosquitoes or representative of distinct biological functions played by CLIPC9 and CLIPA8 (*i.e.* activation of distinct PPOs downstream of CLIPA8 and CLIPC9). Addressing this finding will require more investigation, especially considering the strong reduction in ookinete melanization following ds*CTL4*/ds*CLIPC9* co-silencing. Given its speculative nature, we placed interpretation of these findings in the Discussion on lines 463-77.

Some additional speculation would be appreciated regarding the absence of CLIPC9 cleavage and the production of the p12 product as described in Figure 8. If the p12 band is indicative of an active product that is dependent on upstream CLIPs, could the initial processing of the p30 product be independent of these upstream CLIPs?

We are actively working to obtain the amino acid sequences for the p30 and p12 cleavage products, which may allow us to definitively determine if they derive from a one or multiple cleavage event(s). Four observations lead us to favor the model presented where a single cleavage generates the p30 and p12 fragments: 1) The sizes of the p30 and p12 cleavage products correspond very well with the expected disulfide-linked fragments generated following CLIPC9 proteolysis at its predicted cut site. We have included a sentence in the Results section addressing this point on lines 268-69. 2) The p30 and p12 cleavage products are disulfide linked, as indicated by their failure to separate under non-reducing conditions in Fig. S5. Moreover, running these samples under non-reducing conditions rescues the depletion of CLIPC9-Full 240 min post-*E. coli* challenge suggesting that the disulfide-linked p30 and p12 cleavage products form the full-length protein. A sentence addressing this point has been added to the Results section on lines 276-78. 3) A second proteolytic event generating either fragment would likely produce a third fragment, which was not observed in our analyses. 4) Whole hemolymph westerns performed 20 min after *E. coli* bioparticle injection only revealed the p30 band (Fig. S9), however analyses of concentrated bioparticle pellets revealed that both p30 and p12 fragments were present at this timepoint. This suggested to us that at the 20 min timepoint a low level of full-length CLIPC9 cleavage could result in the p12 fragment being below the limit of detection by whole hemolymph western. Based on these points, we believe that CLIPC9 cleavage at or near its predicted cut site to be the most parsimonious interpretation. A sentence indicating this point has been added to the Results section on lines 278-80. However, it is possible, as the reviewer suggests, that some CLIPC9 cleavage is independent of the upstream CLIP-SPHs. We appreciate this point and have added to the Discussion on lines 527-29 to acknowledge this possibility.

The paragraph containing line 431-454 is interesting and thought provoking. An additional point to consider is that the late-phase immune response where multiple PPOs have been implicated does not involve melanization. Based on Dudzic et al., this might suggest that the effects of CLIPC9 are comparable to Hayan responsible for the blackening reaction (as you describe), leaving the anti-microbial effects of PPOs to potentially be processed by another CLIP.

We thank the reviewer for this interpretation of the data. We agree with this alternative interpretation of CLIPC9’s possible role in parasite darkening, but not oocyst killing and have included a sentence in the Discussion on lines 499-502 highlighting this possibility.

Specific comments

Line 60: Should be corrected to “vector-borne”

We thank reviewer 3 for identifying this typographical error. The correction is now on line 62.
